# Supplementary material for: Nest-site selection and breeding success of passerines in the world’s southernmost forests
Source: PeerJ. 2020 Sep 21;8:e9892. doi: 10.7717/peerj.9892 (PMC7513745; doi:10.7717/peerj.9892)
Supplement: Table S1 — Mean value ± 2 standard error (SE) of habitat characteristics measured in nest plots of five forest-nesting bird species on Navarino Island, Chile, 2014–2017. [file peerj-08-9892-s001.docx]

Supplemental Table S1

Mean value ± 2 standard error (SE) of habitat characteristics measured in nest plots of five forest-nesting bird species on Navarino Island, Chile, 2014-2017.

| **Species (n)** | **Canopy cover (%)** | **Canopy height (m)** | **Understory cover (%)** | **Understory height (cm)** |
| --- | --- | --- | --- | --- |
| *E. albiceps* (22) | 37.8 ± 8.9 | 6.5 ± 1.7 | 39.4 ± 8.5 | 122.26 ± 16.9 |
| *Z. capensis* (33) | 26.8 ± 6.8 | 5.3 ± 1.2 | 45.0 ± 6.7 | 100.1 ± 9.9 |
| *P. patagonicus* (17) | 24.6 ± 10.6 | 10.0 ± 7.8 | 49.8 ± 12.0 | 107.6 ± 19.1 |
| *T. falcklandii* (8) | 46.4 ± 17.0 | 11.1 ± 4.2 | 30.3 ± 17.0 | 95.8 ± 20.6 |
| *A. parulus* (16) | 21.8 ± 11.2 | 4.3 ± 2.4 | 68.0 ± 13.8 | 95.8 ± 20.2 |
