# Supplementary material for: Nest-site selection and breeding success of passerines in the world’s southernmost forests
Source: PeerJ. 2020 Sep 21;8:e9892. doi: 10.7717/peerj.9892 (PMC7513745; doi:10.7717/peerj.9892)
Supplement: Table S2 — Goodness of fit of final models of nest-site selection and daily survival rate, for five forest dwelling passerines on Navarino Island, Chile, 2014–2017. We assessed this with χ2 tests, accepting the model if p > 0.05. [file peerj-08-9892-s002.docx]

Supplemental Table S2

Goodness of fit of final models of nest-site selection and daily survival rate, for five forest dwelling passerines on Navarino Island, Chile, 2014-2017. We assessed this with χ^2^ tests, accepting the model if *p* > 0.05.

| Species | Nest site selection model | | Daily nest survival rate model | |
| --- | --- | --- | --- | --- |
|  | χ^2^ | *p*-value | χ^2^ | *p*-value |
| *Elaenia albiceps* | 4.92 | 0.766 | 286.65 | 0.511 |
| *Zonotrichia capensis* | 8.78 | 0.361 | 162.87 | 1.000 |
| *Phrygilus patagonicus* | 10.95 | 0.205 | 0 | 1.000 |
| *Turdus falcklandii* | ^*^ | ^*^ | 17.78 | 1.000 |
| *Anairetes parulus* | 4.73 | 0.786 | 0 | 1.000 |

^*^ The final model for this species is the null model
