# Supplementary material for: Nest-site selection and breeding success of passerines in the world’s southernmost forests
Source: PeerJ. 2020 Sep 21;8:e9892. doi: 10.7717/peerj.9892 (PMC7513745; doi:10.7717/peerj.9892)
Supplement: Table S3 — Candidate models describing nest-site selection of five forest dwelling passerines on Navarino Island, Chile, 2014–2017. The habitat variables we tested included canopy cover, canopy height, understory cover, and understory height. Models are ranked by AICc, with the best supported model given in bold (i.e., the model with lowest AICc). [file peerj-08-9892-s003.docx]

Supplemental Table S3

Candidate models describing nest-site selection of five forest dwelling passerines on Navarino Island, Chile, 2014-2017. The habitat variables we tested included canopy cover, canopy height, understory cover, and understory height. Models are ranked by AICc, with the best supported model given in bold (i.e., the model with lowest AICc).

*Elaenia albiceps*

| **Model** | **K** | **LL** | **AIC_c_** | **ΔAIC_c_** | **Weight** |
| --- | --- | --- | --- | --- | --- |
| **~ Canopy height** | **2** | **-28.77** | **61.7** | **0.00** | **0.203** |
| ~ Canopy height + understory height | 3 | -28.11 | 62.8 | 1.12 | 0.116 |
| ~ Canopy height + canopy cover | 3 | -28.25 | 63.1 | 1.38 | 0.101 |
| ~ Intercept | 1 | -30.50 | 63.1 | 1.39 | 0.101 |
| ~ Canopy height + canopy cover + understory height | 4 | -27.30 | 63.6 | 1.92 | 0.078 |
| ~ Canopy height + understory cover | 3 | -28.66 | 63.8 | 2.23 | 0.067 |
| ~ Understory height | 2 | -29.82 | 63.9 | 2.24 | 0.066 |
| ~ Canopy height + canopy cover + understory cover | 4 | -27.51 | 64.0 | 2.34 | 0.063 |
| ~ Understory cover | 2 | -30.36 | 65.0 | 3.31 | 0.039 |
| ~ Canopy height + understory height + understory cover | 4 | -28.11 | 65.2 | 3.54 | 0.035 |
| ~ Canopy cover | 2 | -30.50 | 65.3 | 3.58 | 0.034 |
| ~ Canopy height + canopy cover + understory height + understory cover | 5 | -26.86 | 65.3 | 3.60 | 0.034 |
| ~ Canopy cover + understory height | 3 | -29.76 | 66.1 | 4.42 | 0.022 |
| ~ Understory height + understory cover | 3 | -29.81 | 66.2 | 4.52 | 0.021 |
| ~ Canopy cover + understory cover | 3 | -30.25 | 67.1 | 5.38 | 0.014 |
| ~ Canopy cover + understory height + understory cover | 4 | -29.66 | 68.3 | 6.63 | 0.007 |

*Zonotrichia capensis*

| **Model** | **K** | **LL** | **AIC_c_** | **ΔAIC_c_** | **Weight** |
| --- | --- | --- | --- | --- | --- |
| **~ Understory cover** | **2** | **-42.21** | **88.6** | **0.00** | **0.303** |
| ~ Understory cover + understory height | 3 | -41.79 | 90.0 | 1.36 | 0.153 |
| ~ Understory cover + canopy height | 3 | -42.07 | 90.5 | 1.93 | 0.116 |
| ~ Understory cover + canopy cover | 3 | -42.13 | 90.7 | 2.05 | 0.109 |
| ~ Understory cover + canopy height + canopy cover | 4 | -41.65 | 92.0 | 3.36 | 0.056 |
| ~ Understory cover + understory height + canopy height | 4 | -41.71 | 92.1 | 3.48 | 0.053 |
| ~ Understory cover + understory height + canopy cover | 4 | -41.75 | 92.1 | 3.54 | 0.052 |
| ~ Understory height | 2 | -44.24 | 92.7 | 4.06 | 0.040 |
| ~ Intercept | 1 | -45.76 | 93.6 | 4.95 | 0.025 |
| ~ Understory cover + understory height + canopy height + canopy cover | 5 | -41.45 | 93.9 | 5.30 | 0.021 |
| ~ Understory height + canopy cover | 3 | -44.06 | 94.5 | 5.90 | 0.016 |
| ~ Understory height + canopy height | 3 | -44.19 | 94.8 | 6.16 | 0.014 |
| ~ Canopy cover | 2 | -45.36 | 94.9 | 6.30 | 0.013 |
| ~ Canopy height + canopy cover | 3 | -44.42 | 95.2 | 6.62 | 0.011 |
| ~ Canopy height | 2 | -45.59 | 95.4 | 6.76 | 0.010 |
| ~ Understory height + canopy height + canopy cover | 4 | -43.65 | 96.0 | 7.35 | 0.008 |

*Phrygilus patagonicus*

| **Model** | **K** | **LL** | **AIC_c_** | **ΔAIC_c_** | **Weight** |
| --- | --- | --- | --- | --- | --- |
| **~ Understory cover + understory height** | **3** | **-18.84** | **44.5** | **0.00** | **0.283** |
| ~ Understory cover | 2 | -20.85 | 46.1 | 1.61 | 0.127 |
| ~ Understory cover + understory height + canopy cover | 4 | -18.60 | 46.6 | 2.11 | 0.098 |
| ~ Understory cover + understory height + canopy height | 4 | -18.68 | 46.7 | 2.25 | 0.092 |
| ~ Understory height | 2 | -21.41 | 47.2 | 2.72 | 0.073 |
| ~ Understory cover + canopy cover | 3 | -20.23 | 47.3 | 2.79 | 0.070 |
| ~ Understory cover + canopy height | 3 | -20.32 | 47.4 | 2.97 | 0.064 |
| ~ Understory height + canopy cover | 3 | -21.08 | 49.0 | 4.48 | 0.030 |
| ~ Understory cover + understory height + canopy cover + canopy height | 5 | -18.49 | 49.1 | 4.64 | 0.028 |
| ~ Understory cover + canopy cover + canopy height | 4 | -19.88 | 49.1 | 4.66 | 0.028 |
| ~ Intercept | 1 | -23.57 | 49.3 | 4.78 | 0.026 |
| ~ Understory height + canopy height | 3 | -21.25 | 49.3 | 4.83 | 0.025 |
| ~ Canopy cover | 2 | -22.56 | 49.5 | 5.04 | 0.023 |
| ~ Canopy height | 2 | -23.01 | 50.4 | 5.93 | 0.015 |
| ~ Canopy cover + canopy height | 3 | -22.25 | 51.3 | 6.82 | 0.009 |
| ~ Understory height + canopy cover + canopy height | 4 | -20.98 | 51.3 | 6.86 | 0.009 |

*Turdus falcklandii*

| **Model** | **K** | **LL** | **AIC_c_** | **ΔAIC_c_** | **Weight** |
| --- | --- | --- | --- | --- | --- |
| **~ Intercept** | **1** | **-11.09** | **24.5** | **0.00** | **0.339** |
| ~ Understory cover | 2 | -10.67 | 26.3 | 1.79 | 0.139 |
| ~ Canopy cover | 2 | -10.72 | 26.4 | 1.89 | 0.132 |
| ~ Understory height | 2 | -11.02 | 27.0 | 2.49 | 0.098 |
| ~ Canopy height | 2 | -11.05 | 27.0 | 2.56 | 0.094 |
| ~ Understory cover + canopy cover | 3 | -10.56 | 29.1 | 4.64 | 0.033 |
| ~ Understory cover + canopy height | 3 | -10.60 | 29.2 | 4.73 | 0.032 |
| ~ Understory cover + understory height | 3 | -10.66 | 29.3 | 4.85 | 0.030 |
| ~ Canopy cover + canopy height | 3 | -10.66 | 29.3 | 4.85 | 0.030 |
| ~ Understory height + canopy cover | 3 | -10.70 | 29.4 | 4.93 | 0.029 |
| ~ Understory height + canopy height | 3 | -11.01 | 30.0 | 5.55 | 0.021 |
| ~ Understory cover + canopy cover + canopy height | 4 | -10.32 | 32.3 | 7.82 | 0.007 |
| ~ Understory cover + understory height + canopy cover | 4 | -10.55 | 32.7 | 8.27 | 0.005 |
| ~ Understory height + canopy cover + canopy height | 4 | -10.59 | 32.8 | 8.34 | 0.005 |
| ~ Understory cover + understory height + canopy height | 4 | -10.60 | 32.8 | 8.36 | 0.005 |
| ~ Understory cover + understory height + canopy cover + canopy height | 5 | -10.32 | 36.6 | 12.17 | 0.001 |

*Anairetes parulus*

| **Model** | **K** | **LL** | **AIC_c_** | **ΔAIC_c_** | **Weight** |
| --- | --- | --- | --- | --- | --- |
| **~ Understory cover + understory height + canopy height** | **4** | **-16.78** | **43.0** | **0.00** | **0.170** |
| ~ Understory cover | 2 | -19.33 | 43.1 | 0.04 | 0.166 |
| ~ Understory cover + understory height | 3 | -18.22 | 43.3 | 0.27 | 0.148 |
| ~ Understory cover + canopy height | 3 | -18.67 | 44.2 | 1.15 | 0.095 |
| ~ Understory cover + understory height + canopy height + canopy cover | 5 | -16.02 | 44.3 | 1.30 | 0.089 |
| ~ Understory cover + canopy cover | 3 | -19.10 | 45.1 | 2.02 | 0.062 |
| ~ Understory height + canopy height | 3 | -19.31 | 45.5 | 2.45 | 0.050 |
| ~ Understory cover + understory height + canopy cover | 4 | -18.01 | 45.5 | 2.47 | 0.049 |
| ~ Understory cover + canopy height + canopy cover | 4 | -18.11 | 45.7 | 2.67 | 0.045 |
| ~ Intercept | 1 | -22.18 | 46.5 | 3.46 | 0.030 |
| ~ Canopy height | 2 | -21.10 | 46.6 | 3.57 | 0.028 |
| ~ Understory height | 2 | -21.40 | 47.2 | 4.18 | 0.021 |
| ~ Understory height + canopy height + canopy cover | 4 | -18.97 | 47.4 | 4.40 | 0.019 |
| ~ Canopy height + canopy cover | 3 | -20.86 | 48.6 | 5.54 | 0.011 |
| ~ Canopy cover | 2 | -22.16 | 48.7 | 5.70 | 0.010 |
| ~ Understory height + canopy cover | 3 | -21.38 | 49.6 | 6.59 | 0.006 |
