# Supplementary material for: Nest-site selection and breeding success of passerines in the world’s southernmost forests
Source: PeerJ. 2020 Sep 21;8:e9892. doi: 10.7717/peerj.9892 (PMC7513745; doi:10.7717/peerj.9892)
Supplement: Table S4 — Best supported models explaining variability in mean nest daily survival rate (DSR) for each of the five forest-nesting bird species on Navarino Island, Chile, 2014–2017. We report estimate (B) and their 95% confidence interval. [file peerj-08-9892-s004.docx]

Supplemental Table S4

Best supported models explaining variability in mean nest daily survival rate (DSR) for each of the five forest-nesting bird species on Navarino Island, Chile, 2014-2017. We report estimate ($\beta$) and their 95% confidence interval.

| **Coefficients in the best supported model** | ***Elaenia albiceps*** | ***Zonotrichia capensis*** | ***Phrygilus patagonicus*** | ***Turdus falcklandii*** | ***Anairetes parulus*** |
| --- | --- | --- | --- | --- | --- |
| Nest age | - 2.58  (-7.14 – -0.89) | - 8.90  (-18.68 – -2.62) | -1.25  (-2.75 ­– -0.10) |  | -24.94  (-54.35 – 4.48) |
| Nest age^2^ | 3.66  (0.88 – 7.32) | 6.25  (1.40 – 13.64) |  |  | 23.93  (-3.59 – 51.46) |
| Nest stage |  | 1.65  (0.23 – 3.17) |  |  |  |
| Nest height |  |  |  |  | 6.15  (-4.84 – 16.59) |
| Canopy cover | - 0.78  (-1.39 – -0.20) |  |  |  |  |
| Understory height | - 0.85  (-1.49 – -0.24) |  | -0.96  (-2.09 – -0.04) |  |  |
| Understory cover |  |  | -1.62  (-3.77 – -0.28) | -1.13 (-2.68 – -0.26) | 1.12  (-0.09 – 2.33) |
| Concealment | -0.51 (-1.70, 0.13) |  | 1.18  (0.06 – 2.90) |  |  |
| Camera |  | 1.97  (0.20 – 3.61) |  |  |  |
