# Supplementary material for: Nest-site selection and breeding success of passerines in the world’s southernmost forests
Source: PeerJ. 2020 Sep 21;8:e9892. doi: 10.7717/peerj.9892 (PMC7513745; doi:10.7717/peerj.9892)
Supplement: Table S5 — Candidate models describing daily nest survival rate (DSR) of five forest dwelling passerines on Navarino Island, Chile, 2014-2017. In the first stage of model selection, we evaluated temporal parameters. On the second stage, we added habitat parameters to the best-supported model from stage one. On the first stage we tested for nest stage (egg [laying and incubation] vs nestling), day of year (date, linear and quadratic effects), and nest age (linear and quadratic effects). On the second stage, the parameters we tested for included nest coverage (concealment, computed as the mean side [measured at the four cardinal directions] and overhead cover), canopy cover, canopy height, understory cover, understory height, nest height, and ground predator accessibility index (predator index, ranging from 0–2, indicating increasing nest accessibility for a potential ground predator). Models are ranked by AICc, with the best supported model given in bold (i.e., the model with lowest AICc). For each stage of model selection, we present the first 10 candidate models only. [file peerj-08-9892-s005.docx]

Supplemental Table S5

Candidate models describing daily nest survival rate (DSR) of five forest dwelling passerines on Navarino Island, Chile, 2014-2017. In the first stage of model selection, we evaluated temporal parameters. On the second stage, we added habitat parameters to the best-supported model from stage one. On the first stage we tested for nest stage (egg [laying and incubation] vs nestling), day of year (date, linear and quadratic effects), and nest age (linear and quadratic effects). On the second stage, the parameters we tested for included nest coverage (concealment, computed as the mean side [measured at the four cardinal directions] and overhead cover), canopy cover, canopy height, understory cover, understory height, nest height, and ground predator accessibility index (predator index, ranging from 0-2, indicating increasing nest accessibility for a potential ground predator). Models are ranked by AIC_c_, with the best supported model given in bold (i.e., the model with lowest AIC_c_). For each stage of model selection, we present the first 10 candidate models only.

| *Elaenia albiceps* |  |  |  |  |  |
| --- | --- | --- | --- | --- | --- |
| **Model.** All models include season as random effect | **K** | **LL** | **AIC_c_** | **ΔAIC_c_** | **Weight** |
| Stage 1: Temporal variables |  |  |  |  |  |
| **~ Nest age + nest age^2^** | **4** | **-62.40** | **132.9** | **0.00** | **0.151** |
| ~ Intercept | 2 | -64.88 | 133.8 | 0.86 | 0.098 |
| ~ Nest age + nest age^2^ + date | 5 | -62.16 | 134.5 | 1.60 | 0.068 |
| ~ Nest age + nest age^2^ + nest stage | 5 | -62.18 | 134.6 | 1.62 | 0.067 |
| ~ Date | 3 | -64.62 | 135.3 | 2.38 | 0.046 |
| ~ Nest age + nest age^2^ + date + date^2^ | 6 | -61.65 | 135.6 | 2.65 | 0.040 |
| ~ Nest stage | 3 | -64.86 | 135.8 | 2.87 | 0.036 |
| ~ Nest age | 3 | -64.86 | 135.8 | 2.88 | 0.036 |
| ~ Nest age + nest age^2^ + date + nest stage | 6 | -62.03 | 136.3 | 3.41 | 0.027 |
| ~ Date + date^2^ | 4 | -64.23 | 136.6 | 3.66 | 0.024 |
| Stage 2: Habitat variables |  |  |  |  |  |
| **~ Nest age + nest age^2^ + understory height + canopy cover + concealment** | **7** | **-57.79** | **130.0** | **0.00** | **0.046** |
| ~ Nest age + nest.age^2^ + understory height + canopy cover | 6 | -59.02 | 130.3 | 0.37 | 0.038 |
| ~ Nest age + nest.age^2^ + understory height + canopy cover + concealment + canopy height | 8 | -57.15 | 130.8 | 0.83 | 0.030 |
| ~ Nest age + nest.age^2^ + understory height + canopy cover + concealment + nest height | 8 | -57.17 | 130.8 | 0.87 | 0.030 |
| ~ Nest age + nest.age^2^ + understory height + canopy cover + concealment + nest height + canopy height | 9 | -56.14 | 130.9 | 0.95 | 0.029 |
| ~ Nest age + nest.age^2^ + understory height + canopy cover + nest height | 7 | -58.59 | 131.6 | 1.61 | 0.021 |
| ~ Nest age + nest age^2^ + understory height + canopy cover + concealment + understory cover | 8 | -57.70 | 131.9 | 1.94 | 0.018 |
| ~ Nest age + nest age^2^ + understory height + canopy cover + canopy height | 7 | -58.82 | 132.0 | 2.06 | 0.016 |
| ~ Nest age + nest age^2^ + understory height + canopy cover + concealment + predator index | 9 | -56.82 | 132.3 | 2.30 | 0.015 |

| *Zonotrichia capensis* |  |  |  |  |  |
| --- | --- | --- | --- | --- | --- |
| **Model**  All models include season as random effect | **K** | **LL** | **AIC_c_** | **ΔAIC_c_** | **Weight** |
| Stage 1: Temporal variables |  |  |  |  |  |
| **~ Camera + nest age + nest age^2^ + nest stage** | **6** | **-55.97** | **124.2** | **0.00** | **0.215** |
| ~ Camera + nest age + nest age^2^ + nest stage + date | 7 | -55.71 | 125.8 | 1.56 | 0.098 |
| ~ Camera + nest age + nest age^2^ + nest stage + date + date^2^ | 8 | -55.31 | 127.1 | 2.88 | 0.051 |
| ~ Camera + nest age + nest ge^2^ | 5 | -58.56 | 127.3 | 3.09 | 0.046 |
| ~ Camera + nest age + nest age^2^ + date | 6 | -57.95 | 128.2 | 3.97 | 0.030 |
| ~ Camera + nest age + nest stage + date | 6 | -58.54 | 129.4 | 5.14 | 0.016 |
| ~ Camera + nest age + nest age^2^ + date + date^2^ | 7 | -57.56 | 129.5 | 5.28 | 0.015 |
| ~ Camera + nest age + nest stage | 5 | -59.66 | 129.5 | 5.31 | 0.015 |
| ~ Camera + nest age + date | 5 | -59.79 | 129.8 | 5.55 | 0.013 |
| ~ Camera + nest age + nest stage - date + date^2^ | 7 | -57.77 | 129.9 | 5.70 | 0.012 |
| Stage 2: Habitat variables |  |  |  |  |  |
| **~ Camera + nest age + nest age^2^ + nest stage** | **6** | **-55.97** | **124.2** | **0.00** | **0.118** |
| **~** Camera + nest age + nest age^2^ + nest stage + concealment | 7 | -55.85 | 126.1 | 1.86 | 0.046 |
| **~** Camera + nest age + nest age^2^ + nest stage + canopy height | 7 | -55.89 | 126.2 | 1.94 | 0.045 |
| **~** Camera + nest age + nest age^2^ + nest stage + understory height | 7 | -55.95 | 126.3 | 2.05 | 0.042 |
| **~** Camera + nest age + nest age^2^ + nest stage + canopy cover | 7 | -55.97 | 126.3 | 2.09 | 0.041 |
| **~** Camera + nest age + nest age^2^ + nest stage + understory cover | 7 | -55.97 | 126.3 | 2.09 | 0.041 |
| **~** Camera + nest age + nest age^2^ + nest stage + concealment + canopy height | 8 | -55.81 | 128.1 | 3.89 | 0.017 |
| **~** Camera + nest age + nest age^2^ + nest stage + concealment + understory height | 8 | -55.84 | 128.2 | 3.94 | 0.016 |
| **~** Camera + nest age + nest age^2^ + nest stage + concealment + understory cover | 8 | -55.85 | 128.2 | 3.97 | 0.016 |
| **~** Camera + nest age + nest age^2^ + nest stage + concealment + canopy cover | 8 | -55.85 | 128.2 | 3.97 | 0.016 |

| *Phrygilus patagonicus* |  |  |  |  |  |
| --- | --- | --- | --- | --- | --- |
| **Model** | **K** | **LL** | **AIC_c_** | **ΔAIC_c_** | **Weight** |
| Stage 1: Temporal variables |  |  |  |  |  |
| **~ Nest age** | **2** | **-26.78** | **57.6** | **0.00** | **0.075** |
| ~ Nest stage | 2 | -26.92 | 57.9 | 0.28 | 0.065 |
| ~ Intercept | 1 | -27.96 | 57.9 | 0.34 | 0.064 |
| ~ Date | 2 | -27.11 | 58.3 | 0.66 | 0.054 |
| ~ Nest age + nest age^2^ | 3 | -26.12 | 58.3 | 0.72 | 0.053 |
| ~ Date + date^2^ | 3 | -26.31 | 58.7 | 1.09 | 0.044 |
| ~ Nest age + date | 3 | -26.54 | 59.2 | 1.55 | 0.035 |
| ~ Nest stage + date | 3 | -26.60 | 59.3 | 1.68 | 0.033 |
| ~ Nest age + nest stage | 3 | -26.71 | 59.5 | 1.90 | 0.029 |
| ~ Nest age + date + date^2^ | 4 | -25.78 | 59.7 | 2.09 | 0.026 |
| Stage 2: Habitat variables |  |  |  |  |  |
| **~ Nest age + concealment - understory cover - understory height** | **5** | **-22.53** | **55.3** | **0.00** | **0.034** |
| ~ Nest age + concealment + understory cover + understory height + canopy cover + canopy height + nest height | 8 | -20.03 | 56.6 | 1.30 | 0.018 |
| ~ Nest age + concealment + understory cover + understory height + canopy cover + canopy height + nest height  + nest height^2^ | 9 | -19.16 | 57.0 | 1.70 | 0.015 |
| ~ Nest age + concealment + understory cover + understory height + canopy cover | 6 | -22.39 | 57.1 | 1.80 | 0.014 |
| ~ Nest age + concealment + understory cover + understory height + canopy height | 6 | -22.44 | 57.2 | 1.90 | 0.013 |
| ~ Nest age + concealment + understory cover + understory height + nest height | 6 | -22.53 | 57.4 | 2.08 | 0.012 |
| ~ Nest age + concealment + understory cover | 4 | -24.62 | 57.4 | 2.10 | 0.012 |
| ~ Nest age + understory cover + understory height | 4 | -24.70 | 57.5 | 2.27 | 0.011 |
| ~ Nest age | 2 | -26.78 | 57.6 | 2.33 | 0.011 |
| ~ Concealment + understory cover + understory height | 4 | -24.84 | 57.8 | 2.55 | 0.010 |

| *Turdus falcklandii* |  |  |  |  |  |
| --- | --- | --- | --- | --- | --- |
| **Model** | **K** | **LL** | **AIC_c_** | **ΔAIC_c_** | **Weight** |
| Stage 1: Temporal variables |  |  |  |  |  |
| **~ Nest age + nest age^2^ + nest stage** | **4** | **-7.04** | **22.7** | **0.00** | **0.258** |
| ~ Nest stage + stage | 3 | -8.97 | 24.3 | 1.63 | 0.114 |
| ~ Nest age + nest age^2^ | 3 | -9.01 | 24.4 | 1.69 | 0.111 |
| ~ Nest stage | 2 | -10.36 | 24.9 | 2.21 | 0.085 |
| ~ Nest age + nest age^2^ + nest stage + date | 5 | -7.03 | 25.0 | 2.30 | 0.081 |
| ~ Nest stage + date | 3 | -9.41 | 25.2 | 2.50 | 0.074 |
| ~ Nest age + nest stage + date | 4 | -8.81 | 26.2 | 3.54 | 0.044 |
| ~ Nest age + nest age^2^ + date | 4 | -8.87 | 26.3 | 3.66 | 0.041 |
| ~ Intercept | 1 | -12.40 | 26.9 | 4.17 | 0.032 |
| ~ Date | 2 | -12.24 | 28.7 | 5.98 | 0.013 |
| Stage 2: Habitat variables |  |  |  |  |  |
| **~ Understory cover** | **2** | **-8.89** | **22.0** | **0.00** | **0.060** |
| ~ Nest age + nest age^2^ + nest stage | 4 | -7.04 | 22.7 | 0.72 | 0.042 |
| ~ Understory cover + nest age + nest age^2^ | 4 | -7.08 | 22.8 | 0.80 | 0.040 |
| ~ Understory cover + nest stage | 3 | -8.45 | 23.3 | 1.30 | 0.031 |
| ~ Understory cover + concealment | 3 | -8.57 | 23.5 | 1.54 | 0.028 |
| ~ Nest age + nest age^2^ + canopy height | 4 | -7.64 | 23.9 | 1.93 | 0.023 |
| ~ Nest stage + canopy height | 3 | -8.78 | 23.9 | 1.97 | 0.022 |
| ~ Understory cover + nest age | 3 | -8.82 | 24.0 | 2.03 | 0.022 |
| ~ Understory cover + canopy height | 3 | -8.83 | 24.0 | 2.07 | 0.021 |
| ~ Understory cover + understory height | 3 | -8.89 | 24.1 | 2.18 | 0.020 |

| *Anairetes parulus* |  |  |  |  |  |
| --- | --- | --- | --- | --- | --- |
| **Model** | **K** | **LL** | **AIC_c_** | **ΔAIC_c_** | **Weight** |
| Stage 1: Temporal variables |  |  |  |  |  |
| **~ Nest age + nest age^2^** | **3** | **-12.65** | **31.4** | **0.00** | **0.366** |
| ~ Nest age + nest age^2^ + date + date^2^ | 5 | -11.16 | 32.7 | 1.24 | 0.197 |
| ~ Nest age + nest age^2^ + date | 4 | -12.63 | 33.49 | 2.06 | 0.131 |
| ~ Nest age + nest age^2^ + date + date^2^ + nest stage | 6 | -10.87 | 34.23 | 2.81 | 0.090 |
| ~ Nest age + nest age^2^ + date + nest stage | 5 | -12.35 | 35.05 | 3.62 | 0.060 |
| ~ Intercept | 1 | -18.91 | 39.85 | 8.42 | 0.005 |
| ~ Nest stage | 2 | -18.87 | 41.81 | 10.38 | 0.002 |
| ~ Date | 2 | -18.87 | 41.81 | 10.39 | 0.002 |
| ~ Nest age | 2 | -18.91 | 41.90 | 10.47 | 0.002 |
| ~ Date + date^2^ | 3 | -18.05 | 42.24 | 10.81 | 0.002 |
| Stage 2: Habitat variables |  |  |  |  |  |
| **~ Nest age + nest age^2^ + nest height + understory cover** | **5** | **-9.42** | **29.2** | **0** | **0.145** |
| ~ Nest age + nest age^2^ + nest height + nest height^2^ + understory cover | 6 | -8.37 | 29.2 | 0.03 | 0.143 |
| ~ Nest age + nest age^2^ + nest height | 4 | -11.04 | 30.3 | 1.11 | 0.083 |
| ~ Nest age + nest age^2^ + nest height + nest height^2^ | 5 | -10.11 | 30.6 | 1.37 | 0.073 |
| ~ Nest age + nest age^2^ + nest height + nest height^2^ + understory cover + canopy cover | 7 | -8.29 | 31.2 | 2.04 | 0.052 |
| ~ Nest age + nest age^2^ + understory height + canopy cover | 5 | -10.51 | 31.4 | 2.17 | 0.049 |
| ~ Nest age + nest age^2^ | 3 | -12.65 | 31.4 | 2.23 | 0.048 |
| ~ Nest age + nest age^2^ + understory cover + canopy cover | 5 | -10.62 | 31.6 | 2.39 | 0.044 |
| ~ Nest age + nest age^2^ + nest height + canopy cover | 5 | -10.62 | 31.6 | 2.40 | 0.044 |
| ~ Nest age + nest age^2^ + understory cover + understory height + canopy cover | 6 | -9.88 | 32.3 | 3.06 | 0.032 |
